# Survival and Axonal Regeneration of Retinal Ganglion Cells in a Mouse Optic Nerve Crush Model After a Cell-Based Intravitreal Co-Administration of Ciliary Neurotrophic Factor and Glial Cell Line-Derived Neurotrophic Factor at Different Post-Lesion Time Points

**DOI:** 10.3390/cells14090643

**Published:** 2025-04-28

**Authors:** Yue Hu, Lynn Michelle Grodzki, Udo Bartsch

**Affiliations:** Department of Ophthalmology, Experimental Ophthalmology, University Medical Center Hamburg-Eppendorf, 20246 Hamburg, Germany; mia.huy@outlook.com (Y.H.); l.grodzki@uke.de (L.M.G.)

**Keywords:** ciliary neurotrophic factor, glial cell line-derived neurotrophic factor, neuroprotection, neurotrophic factor, optic neuropathy, regeneration

## Abstract

We recently showed, in a mouse optic nerve crush model, that a sustained cell-based intravitreal administration of ciliary neurotrophic factor (CNTF) and glial cell line-derived neurotrophic factor (GDNF) synergistically slowed the lesion-induced degeneration of retinal ganglion cells (RGCs), resulting in the presence of approximately 35% viable RGCs eight months after the lesion. However, the combinatorial neuroprotective treatment was initiated shortly after the lesion. To mimic a more clinically relevant situation, we co-administered both factors either three or five days after an intraorbital nerve crush when approximately 35% or 57% of the RGCs were degenerated, respectively. Analyses of the retinas at different time points after the lesion consistently revealed the presence of significantly more surviving RGCs in retinas co-treated with CNTF and GDNF than in retinas treated with either factor alone. For example, when the neurotrophic factors were administered five days after the nerve crush and the animals were analyzed two months after the lesion, retinas co-treated with CNTF and GDNF contained approximately 40% of the RGCs present at the start of treatment. In comparison, monotherapy with either CNTF or GDNF protected only about 15% or 10% of the RGCs present at baseline, respectively. The number of regenerating axons in the distal nerve stumps was similar in CNTF- and CNTF/GDNF-treated animals, despite the significantly higher number of rescued RGCs in the latter group. These findings have potential implications for studies aimed at developing neuroprotective treatments for optic neuropathies such as glaucoma.

## 1. Introduction

Retinal ganglion cells (RGCs) are projection neurons that transmit visual information from the retina to the brain. Based on morphological, physiological and molecular criteria, RGCs can be divided into more than 40 subtypes [1,2,3]. Of note, RGC subtypes differ significantly in their ability to survive under various pathological conditions and their ability to regrow axons following injury [3,4,5,6,7]. The progressive loss of this heterogeneous group of projection neurons is a common feature of several retinal diseases such as traumatic, ischemic, inflammatory or glaucomatous optic neuropathies [8,9,10]. Treatments for these diseases are either not available or of limited efficacy. For example, glaucoma is among the most common neurodegenerative diseases and a leading cause of irreversible blindness [11], with an estimated number of more than 110 million patients worldwide in 2040 [12]. Elevated intraocular pressure (IOP) is considered the major risk factor for glaucoma, and lowering IOP is the only clinically proven treatment to slow disease progression. However, despite effective IOP-lowering treatments, glaucoma progresses in a significant proportion of patients. In addition, glaucoma can develop in patients whose IOP is within the physiological range and, conversely, a significant number of people with elevated IOP never develop glaucoma [13,14,15].

Neuroprotection aims to slow or halt the progressive degeneration of RGCs and is therefore being investigated as a potential treatment option for glaucoma and other optic neuropathies [16,17,18,19]. Preclinical work has led to the identification of several neurotrophic factors (NTFs) that promote RGC survival in different animal models of RGC loss, such as ciliary neurotrophic factor (CNTF) [20,21,22,23,24] and glial cell line-derived neurotrophic factor (GDNF) [25,26,27,28,29,30]. CNTF has additionally been shown to stimulate long-distance regeneration of injured RGC axons [21,31,32,33].

GDNF is the founding member of the GDNF family ligands. GDNF first binds to membrane-bound or soluble GDNF family receptor α1 (GFRα1). The GDNF-GFRα1 complex activates the receptor tyrosine kinase rearranged during transfection (RET) and other signaling inducing receptors, such as the neural cell adhesion molecule (NCAM) or syndercan-3, which trigger various signaling pathways, including the mitogen-activated protein kinase/extracellular signal-regulated kinase (MAPK/ERK), phosphatidylinositol 3-kinase (PI3K)/Akt and src family kinase pathways [34,35,36,37,38]. CNTF belongs to the interleukin-6 (IL6) family of cytokines. CNTF first binds to the non-signaling membrane-bound or soluble CNTF receptor α (CNTFR α) or IL6 receptor α (IL6R α). The heterodimer then recruits the signal transducing receptors leukemia inhibitory factor receptor β (LIFRβ) and glycoprotein 130 (gp130). The signaling pathways triggered by the tripartite CNTF receptor complex include the Janus kinase/signal transducer and activator of the transcription (JAK/STAT), MAPK/ERK and PI3/Akt pathways [39,40,41,42,43,44].

Co-administration of two or more NTFs may support the survival of damaged RGCs more effectively than the corresponding neuroprotective monotherapies, as different NTFs may signal through different receptors to trigger different pro-survival signaling pathways and/or enhance the activation of shared pro-survival signaling pathways. We have previously tested the efficacy of a combinatorial neuroprotective treatment in a mouse optic nerve crush (ONC) model by continuously delivering of CNTF and GDNF to the retina via intravitreal transplantations of lentivirally modified neural stem cell clones [31,45]. These experiments indeed showed that these two NTFs synergistically protected RGCs from lesion-induced cell death in the long term. In retinas co-treated with CNTF and GDNF, around 35% of the RGCs were still viable 8 months after the lesion, compared to only 7% in retinas treated with either CNTF or GDNF alone [31]. Other NTF combinations that have been shown to promote RGC survival in a cooperative, additive or synergistic manner include brain-derived neurotrophic factor (BDNF) and GDNF; BDNF and neurturin; BDNF and CNTF; and BDNF, fibroblast growth factor 2 and neurotrophin-3 [46,47,48,49].

In our and most other preclinical studies, neuroprotective treatments were started at or even before the time when ganglion cell degeneration was induced. However, in terms of potential clinical applications, it is of greater interest to investigate the outcome of neuroprotective treatments initiated at a time when a proportion of RGCs are already degenerated. Therefore, we transplanted the NTF-expressing NSCs some time after an intraorbital ONC and determined the number of surviving RGCs at different time points after the lesion in retinas simultaneously treated with CNTF and GDNF or with each factor alone. The extent of axonal regeneration was assessed by analyzing the length and number of axons extending beyond the lesion site.

## 2. Materials and Methods

The animals used in this study were maintained on a C57BL/6J genetic background and obtained from the Animal Facility of the University Medical Center Hamburg-Eppendorf (Hamburg, Germany). All animal experiments were approved by the Animal Care Committees of the University and the City of Hamburg and were performed in accordance with the guidelines of the ARVO Statement for the Use of Animals in Ophthalmic and Vision Research.

Neural stem cells (NSCs) were isolated from 14-day-old C57BL/6J mouse embryos and transduced with lentiviral vectors encoding either the fluorescent reporter protein Venus (referred to as control-NSCs), or CNTF and the reporter protein Venus (referred to as CNTF-NSCs) or GDNF and the reporter protein enhanced green fluorescent protein (eGFP; referred to as GDNF-NSCs) under the control of a cytomegalovirus enhancer/chicken β-actin promoter (CAG), as previously described [45,50,51].

To generate clonal cell lines with high levels of transgene expression, cells were subjected to multiple rounds of spinoculation, each followed by clonal expansion of the cells with the highest expression of the fluorescent reporter protein. We have shown in previous studies that the CNTF-NSC and GDNF-NSC clones used in this study promote the survival of axotomized RGCs with similar efficacy [31,45]. Detailed characterization of the cell lines showed stable transgene expression in undifferentiated and neurally differentiated cultures [31,45,52]. For intravitreal cell transplantation experiments, subconfluent control-NSC, CNTF-NSC and GDNF-NSC cultures were detached from the culture substrate using Accutase (Life Technologies, Darmstadt, Germany) and resuspended in phosphate-buffered saline (PBS; pH 7.4) at a density of 3.8 × 10^5^ cells/µL. For co-administration experiments of CNTF and GDNF, a 1:1 mixture of the CNTF-NSC and GDNF-NSC clone (hereafter referred to as CNTF/GDNF-NSCs) was prepared at the same final cell density.

Optic nerves were crushed intraorbitally as described [52,53,54]. Briefly, adult (at least two months old) mice were anesthetized, and optic nerves were crushed with watchmaker’s forceps for 15 s 0.5–1.0 mm distal to the optic disc. Criteria for a successful optic nerve crush (ONC) included loss of the pupillary light reflex and the absence of retinal hemorrhages. Intravitreal transplantations of NSCs were performed either 3 or 5 days after the ONC. Using a fine glass micropipette attached to a syringe, 2 µL of vitreous fluid was aspirated and the same volume of the different cell suspensions was slowly injected into the vitreous cavity. Immediately after the ONC or the cell transplantation, eyes were treated with antibiotic eye drops (OFTAQUIX^®^; Santen GmbH, Munich, Germany) and eye gel with wound healing and moisturizing compounds (Corneregel^®^; Bausch + Lomb Incorporated, Berlin, Germany).

Survival, differentiation and transgene expression of transplanted NSCs were analyzed 56 days after the ONC in animals that had received injections of CNTF/GDNF-NSCs 5 days post-lesion (dpl). Animals were euthanized and enucleated, a portion of the cornea was removed and the eyes were fixed in 4% paraformaldehyde (PFA) for 15 min before the lenses were carefully removed. After further fixation for 15 min and blocking for 1 h, lenses with attached donor cells were simultaneously immunostained with antibodies against CNTF (1:100; Santa Cruz Biotechnology, Inc., Dallas, TX, USA) and GDNF (1:50; R&D Systems, Inc., Minneapolis, MN, USA) to analyze the expression of the neuroprotective factors, or with antibodies against glial fibrillary acidic protein (GFAP,1:500; Agilent Technologies, Inc., Santa Clara, CA, USA) and class III β-tubulin (β-TUB III, 1:1000; Sigma-Aldrich, St. Louis, MO, USA) to monitor the differentiation of the donor cells. Primary antibodies were detected with appropriate Cy3- or Cy5-conjugated secondary antibodies (all 1:200; BIOZOL Diagnostica Vertrieb GmbH, Eching, Germany). Some lenses were additionally stained with anti-eGFP antibodies (1:100; R&D Systems) and Cy2-conjugated secondary antibodies (1:200; BIOZOL Diagnostica Vertrieb GmbH) to enhance the Venus or eGFP fluorescence of the donor cells. Cell nuclei were stained with 4′,6-diamidino-2-phenylindole (DAPI, 1:2000; Sigma-Aldrich). For imaging, lenses were pinned to the bottom of silicone-coated Petri dishes using insect needles, fixed again in 4% PFA for 30 min and immersed in PBS. Z-stack images of the donor cell layers were taken with a confocal microscope using a long-working-distance water immersion objective (Olympus FV 1000, Olympus Deutschland GmbH, Hamburg, Germany).

Retinal flatmounts were prepared as described elsewhere [52] and incubated overnight with antibodies against brain-specific homeobox/POU protein 3A (BRN-3A; 1:200; Santa Cruz Biotechnology), followed by Cy3-conjugated secondary antibodies and DAPI. The total RGC population and different RGC subtypes are unevenly distributed in the mouse retina [55,56,57]. In order to determine RGC densities in a retinal area as large and representative as possible, 5 consecutive images were taken from the optic disc to the periphery of the dorsal, ventral, temporal and nasal quadrants of the flatmounts using an epifluorescence microscope (OLYMPUS IX51, Olympus Deutschland GmbH) and imaging software (cellSens Entry version 1.4, Olympus Deutschland GmbH), covering a total area of 1.91 mm^2^. The BRN-3A-positive RGCs were counted by a blinded observer using Photoshop CC 2019 version 20.0.5 (Adobe Inc., San Jose, CA, USA), and the density of RGCs was calculated. RGC densities in animals that received neither cell transplantations nor an ONC were determined as a reference. RGC densities in eyes without transplanted NSCs three and five days after ONC were determined as another reference. RGC densities in eyes that had received transplantation of control-, CNTF-, GDNF- or CNTF/GDNF-NSCs either three or five days after ONC were determined 14, 28 and 56 days after nerve injury. To assess retinal structure in treated animals, eyes grafted with control-NSCs or CNTF/GDNF-NSCs (n = 3 for each treatment) were enucleated 56 days after the nerve lesion and fixed overnight in 4% PFA. Eyes were cryoprotected, frozen and sectioned at a thickness of 25 µm using a cryostat (CM1950, Leica, Wetzlar, Germany). Sections were blocked in PBS containing 0.1% bovine serum albumin and 0.3% Triton X-100, incubated with antibodies against BRN-3A overnight at room temperature, washed in PBS, incubated with Cy3-conjugated secondary antibodies, stained with DAPI and mounted on slides. Retinal sections from untreated wild-type mice (n = 3) were processed in parallel as a reference. Micrographs of flatmounts and sections for qualitative documentation were taken with an Axio Observer Z1 microscope equipped with an ApoTome.2 (Carl Zeiss AG, Oberkochen, Germany).

Axonal regeneration was assessed 28 days after ONC in animals that had received intravitreal injections of control-, CNTF-, GDNF- or CNTF/GDNF-NSCs 5 days after the nerve lesion. One day before euthanasia, RGC axons were anterogradely traced by intravitreal injections of 1.5 µL of a saturated solution of biotin-N-hydroxysuccinimide ester (Sigma-Aldrich) dissolved in dimethylformamide (Carl Roth GmbH + Co. KG, Karlsruhe, Germany) and ethanol [54,58]. The next day, eyes with attached optic nerves were immersion-fixed in 4% PFA, cryoprotected, embedded in Tissue-Tek^®^ O.C.T. Compound (Sakura Finetek USA Inc., Torrance, CA, USA) and frozen. Optic nerves were cut longitudinally at a thickness of 25 µm, incubated with Cy3-conjugated streptavidin (1:200; Jackson ImmunoResearch Laboratories, West Grove, PA, USA) overnight at room temperature, stained with DAPI and mounted on slides. Three optic nerve sections containing the longest regrown axons distal to the lesion site were selected from each animal to analyze axonal regeneration. The length of the longest regrown axon in the distal nerve stump was determined using ZEN 2.1 software (Carl Zeiss AG). For animals treated with CNTF- and CNTF/GDNF-NSCs, the width of nerve sections was measured every 100 µm, starting from 500 µm distal to the lesion site. The number of anterogradely labelled axons at each position was determined using Photoshop CC (Adobe Inc.), and the number of regenerated axons per 100 µm of nerve width was calculated.

Statistical analyses were performed using GraphPad Prism 9 (GraphPad Software, San Diego, CA, USA). RGC densities in eyes with uninjured optic nerves and eyes 3 or 5 days after ONC were compared with a one-way ANOVA. The effect of the different neuroprotective treatments and the delay between ONC and treatment start on RGC survival was analyzed with a two-way ANOVA. The lengths of the longest regrown axons and the number of regrown axons at different positions distal to the lesion site were compared with a one-way ANOVA and a two-way repeated measures ANOVA, respectively. All ANOVAs were followed by a Bonferroni post-hoc test. A *p* value less than 0.05 was considered statistically significant.

## 3. Results

### 3.1. Lesion-Induced Progression of Retinal Ganglion Cell Loss

**Figure 1:**
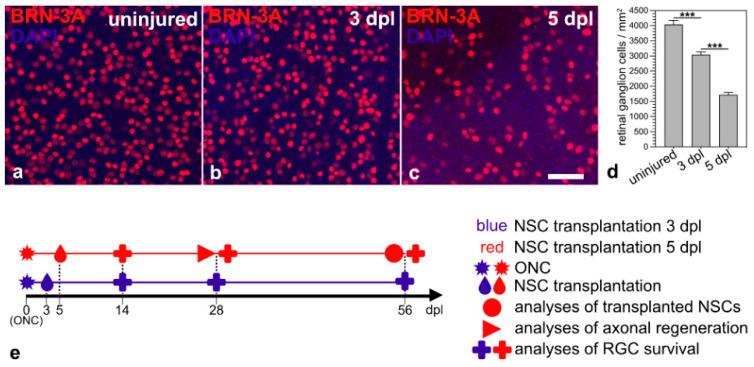
Time course of RGC loss induced by an intraorbital optic nerve crush. Qualitative (**a**–**c**) and quantitative (**d**) analyses of retinal flatmounts 3 (**b**,**d**) and 5 days (**c**,**d**) after ONC revealed a moderate and pronounced loss of BRN-3A-positive RGCs, respectively, when compared to retinas with uninjured optic nerves (**a**,**d**). Each bar in d represents the mean value (±SEM) of six animals. ***, *p* < 0.001 according to a one-way ANOVA followed by a Bonferroni post-hoc test. Schematic representation of the experimental design ((**e**); see Results for details). BRN-3A, brain-specific homeobox/POU domain protein 3A; dpl, days post-lesion; NSC, neural stem cell; ONC, optic nerve crush; RGC, retinal ganglion cell. Scale bar in (**c**) for (**a**–**c**): 50 µm.

The aim of this study was to evaluate the outcome of a neuroprotective treatment initiated at an advanced stage of RGC degeneration. Therefore, we first evaluated the progression of RGC loss in animals that received an ONC but no intravitreal cell transplantation. RGC densities in animals that received neither an ONC nor intravitreal cell transplantations were determined as a reference. Retinas with uninjured optic nerves contained 4040 ± 132 BRN-3A-positive RGCs/mm^2^ (mean ± SEM; n = 6; Figure 1a,d). Animals with intraorbitally injured optic nerves, in comparison, contained 3044 ± 91 RGCs/mm^2^ three days after the lesion (Figure 1b,d) and 1718 ± 82 RGCs/mm^2^ five days after the lesion (Figure 1c,d). Thus, we observed a lesion-induced loss of BRN-3A-positive RGCs of approximately 25% and 57% 3 dpl and 5 dpl, respectively. We chose the two post-lesion time points to start the neuroprotective treatments; analyzed the fate of the grafted NSCs 56 dpl; quantified RGC survival 14, 28 and 56 dpl; and assessed axonal regeneration 28 dpl (Figure 1e).

### 3.2. Analyses of Transplanted NSCs

**Figure 2:**
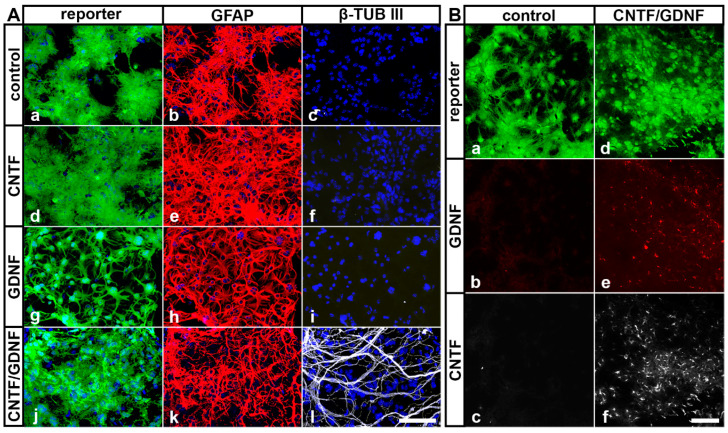
(**A**) Survival and differentiation of intravitreally transplanted NSCs. Analyses of experimental eyes 51 days after cell transplantation revealed the presence of reporter protein-positive donor cells on the posterior surface of the lenses (**A**,**B**). While all control-NSCs (**Aa**–**Ac**), CNTF-NSCs (**Ad**–**Af**) and GDNF-NSCs (**Ag**–**Ai**) differentiated into GFAP-positive astrocytes, a fraction of CNTF/GDNF-NSCs (**Aj**–**Al**) additionally differentiated into β-TUB III–positive neurons. (**B**) Transgene expression in intravitreally transplanted NSCs. Control-NSCs expressed the reporter protein Venus (**Ba**) but not GDNF (**Bb**) or CNTF (**Bc**). CNTF/GDNF-NSCs co-expressed the reporter proteins eGFP or Venus (**Bd**) and GDNF (**Be**) or CNTF (**Bf**), respectively. CNTF, ciliary neurotrophic factor; eGFP, enhanced green fluorescent protein; GDNF, glial cell line-derived neurotrophic factor; GFAP, glial fibrillary acidic protein; NSCs, neural stem cells; β-TUB III, class III β-tubulin. Scale bars: 100 µm.

In our previous studies, we have shown that intravitreally grafted NSCs do not integrate into the host retinas but rather adhere to the posterior poles of the lenses or the vitreal surface of the retinas [31,45,50,52,59]. To evaluate cell viability, neural differentiation and transgene expression in NSCs clones transplanted intravitreally 5 days after ONC, we isolated the lenses 51 days after the cell transplantation and analyzed the attached donor cells by immunocytochemistry. All grafted NSC clones survived in the vitreous cavity for at least 51 days, as indicated by the presence of cells expressing the fluorescent reporter proteins eGFP or Venus (Figure 2). Virtually all control-NSCs, CNTF-NSCs and GDNF-NSCs differentiated into GFAP-positive astrocytes (Figure 2(Ab), Figure 2(Ae) and Figure 2(Ah), respectively). While the majority of CNTF/GDNF-NSCs also differentiated into astrocytes (Figure 2(Ak)), some gave rise to β-TUB III-positive neurons (Figure 2(Al)). Furthermore, cells derived from the CNTF/GDNF-NSCs expressed either CNTF (Figure 2(Bf)) or GDNF (Figure 2(Be)) for at least 51 days after transplantation, whereas astrocytes derived from the control-NSCs expressed neither GDNF (Figure 2(Bb)) nor CNTF (Figure 2(Bc)).

### 3.3. Retinal Ganglion Cell Survival

We next analyzed the impact of the delay between the ONC and the start of the neuroprotective treatment on the extent and progression of RGC loss. To this end, we determined the density of BRN-3A-positive RGCs in flatmounted retinas 14, 28 and 56 dpl in animals that had received intravitreal NSC transplantations either 3 or 5 days after the nerve lesion (Figure 1e).

**Figure 3:**
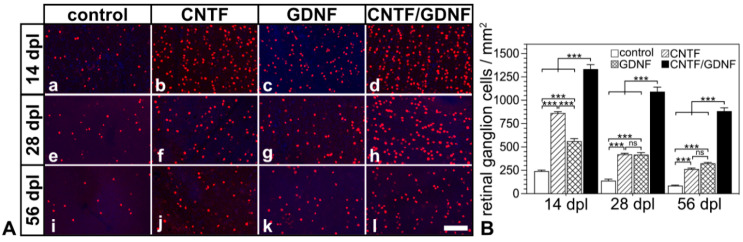
(**A**) Representative micrographs of retinal flatmounts from animals that had received NSC transplantations 3 days after ONC. The density of BRN-3A-positive RGCs in CNTF-treated (**Ab**,**Af**,**Aj**) and GDNF-treated (**Ac**,**Ag**,**Ak**) retinas was significantly higher than in control retinas (**Aa**,**Ae**,**Ai**) 14, 28 and 56 dpl. Note the significantly higher number of surviving RGCs at all post-lesion time points in retinas co-treated with CNTF and GDNF (**Ad**,**Ah**,**Al**). (**B**) Quantitative analysis of RGC survival. Each bar represents the mean value (±SEM) of six animals. ns: not significant; ***: *p* < 0.001 according to a two-way ANOVA followed by a Bonferroni post-hoc test. BRN-3A, brain-specific homeobox/POU domain protein 3A; CNTF, ciliary neurotrophic factor; dpl, days post-lesion; GDNF, glial cell line-derived neurotrophic factor; NSC, neural stem cell; ONC, optic nerve crush; RGCs, retinal ganglion cells. Scale bar: 100 µm.

Flatmounted retinas from animals that had received transplantations of CNTF-, GDNF- or CNTF/GDNF-NSCs 3 days after nerve injury contained markedly more BRN-3A-positive RGCs than retinas from animals with grafted control-NSCs at all analysis time points (Figure 3A). Quantitative analysis confirmed significantly higher RGC densities in CNTF-, GDNF- or CNTF/GDNF-treated retinas than in control retinas 14, 28 and 56 dpl (Figure 3B). While eyes with transplanted control-NSCs contained 242.0 ± 10.1 (mean ± SEM), 139.3 ±16.6 and 86.3 ± 4.9 RGCs/mm^2^; CNTF-treated retinas contained 862.7 ± 15.1, 420.3 ± 12.2, and 263.7 ± 9.9 RGCs/mm^2^; GDNF-treated retinas contained 561.0 ± 27.7, 416.3 ± 21.4 and 324.8 ± 8.0 RGCs/mm^2^; and CNTF/GDNF-treated retinas contained 1333.7 ± 47.9, 1092.0 ± 48.6 and 883.0 ± 35.9 RGCs/mm^2^ at 14, 28 and 56 days post-lesion, respectively. Notably, the combined administration of CNTF and GDNF promoted RGC survival significantly more effectively than the administration of either neuroprotective factor alone. At the 56 dpl time point, for example, CNTF/GDNF-treated retinas contained 3.3-fold more RGCs than CNTF-treated retinas and 2.7-fold more RGCs than GDNF-treated retinas (Figure 3B).

**Figure 4:**
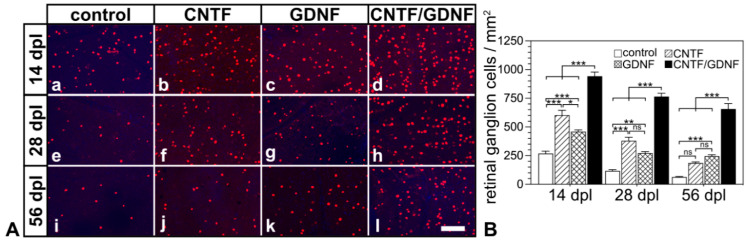
(**A**) Representative micrographs of retinal flatmounts from animals that had received NSC transplantations 5 days after ONC. The density of BRN-3A-positive RGCs in CNTF-treated (**Ab**,**Af**,**Aj**) and GDNF-treated (**Ac**,**Ag**,**Ak**) retinas was significantly higher than in control retinas (**Aa**,**Ae**,**Ai**) 14, 28 and 56 dpl. Note the significantly higher number of surviving RGCs at all post-lesion time points in retinas co-treated with CNTF and GDNF (**Ad**,**Ah**,**Al**). (**B**) Quantitative analysis of RGC survival. Each bar represents the mean value (±SEM) of six animals. ns: not significant; *: *p* < 0.05; **: *p* < 0.01; ***: *p* < 0.001 according to a two-way ANOVA followed by a Bonferroni post-hoc test. BRN-3A, brain-specific homeobox/POU domain protein 3A; CNTF, ciliary neurotrophic factor; dpl, days post-lesion; GDNF, glial cell line–derived neurotrophic factor; NSC, neural stem cell; ONC, optic nerve crush; RGCs, retinal ganglion cells. Scale bar: 100 µm.

Basically similar results were observed when the treatment was started 5 days after the nerve lesion (Figure 4). With the sole exception of retinas that were treated with CNTF 5 dpl and analyzed 56 dpl, RGC densities were significantly higher in retinas treated with the neuroprotective factor-expressing NSCs than in retinas treated with the control-NSC clone (Figure 4B). Specifically, control retinas contained 267.8 ± 20.9, 117.5 ± 10.0 and 64.3 ± 3.6 RGCs/mm^2^; CNTF-treated retinas contained 602.2 ± 42.8, 379.3 ± 30.4, and 186.0 ± 9.5 RGCs/mm^2^; GDNF-treated retinas contained 458.8 ± 15.2, 272.2 ± 13.2 and 245.7 ± 11.7 RGCs/mm^2^; and CNTF/GDNF-treated retinas contained 942.5 ± 36.5, 765.0 ± 29.4 and 657.2 ± 46.1 RGCs/mm^2^ at 14, 28 and 56 days post-lesion, respectively.

Notably, RGC densities in retinas co-treated with CNTF and GDNF were higher in all experimental groups than theoretically expected if both neurotrophic factors would promote RGC survival in an additive manner (Supplementary Table S1). The results show that the co-administration of CNTF and GDNF promoted RGC survival synergistically, regardless of whether the cell transplantations were performed 3 or 5 days after ONC.

A comparison of eyes that had received injections of control-NSCs either 3 dpl or 5 dpl revealed similar RGC densities at all analysis time points (Figure S1). Furthermore, RGC densities were significantly lower in retinas in which the treatment with the different neuroprotective factors was started 5 days after the ONC than in retinas in which the treatment was started 3 days after the ONC, except for CNTF-treated retinas at the 28 days and 56 days post-lesion time points (Figure S1). However, all treatments initiated 5 dpl rescued a higher percentage of the RGC population present at the time of NTF administration than the treatments initiated 3 dpl (Figure S2).

Analysis of retinal sections from animals that were treated with CNTF/GDNF-NSCs 5 days post-lesion and sacrificed 56 days post-lesion confirmed the presence of numerous viable BRN-3A-positive RGCs (Figure S3). In comparison, hardly any RGCs were present in retinas treated with control-NSCs. The overall morphology of the retinas was not adversely affected by the treatment (Figure S3).

### 3.4. Axonal Regeneration

**Figure 5:**
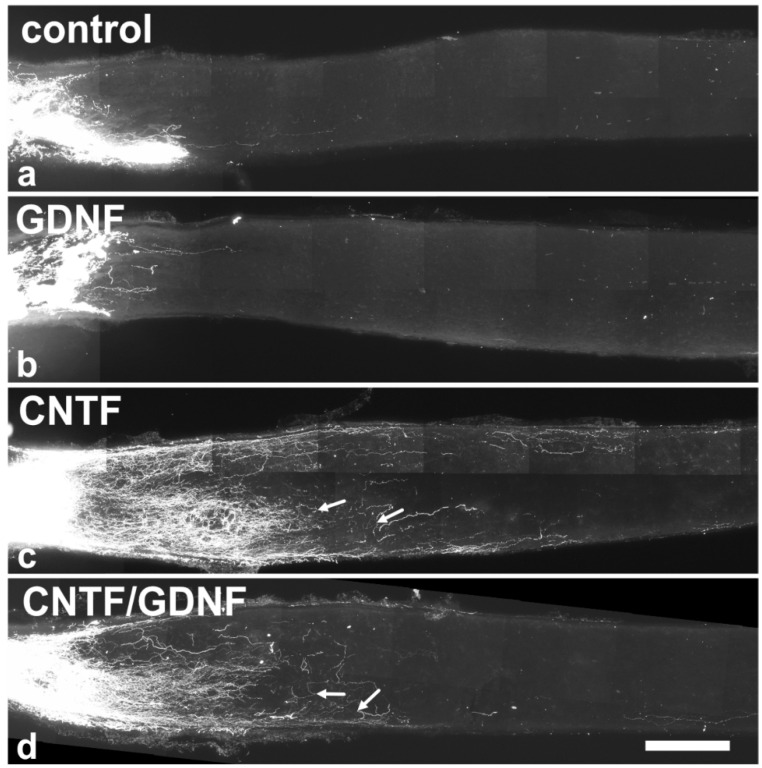
Axonal regeneration in animals that had received NSC transplantations 5 days after ONC. Analyses of animals with grafted control-NSCs (**a**) or GDNF-NSCs (**b**) 28 dpl revealed the presence of only a few regrown axons extending only a short distance beyond the lesion site. Animals with grafted CNTF-NSCs (**c**) or CNTF/GDNF-NSCs (**d**), in comparison, contained significantly more and longer regrown axons in the distal nerve stumps. Axons displayed an irregular course, and some made U-turns (arrows in (**c**,**d**)). CNTF, ciliary neurotrophic factor; dpl, days post-lesion; GDNF, glial cell line-derived neurotrophic factor; NSC, neural stem cell; ONC, optic nerve crush. Scale bar: 200 µm.

**Figure 6:**
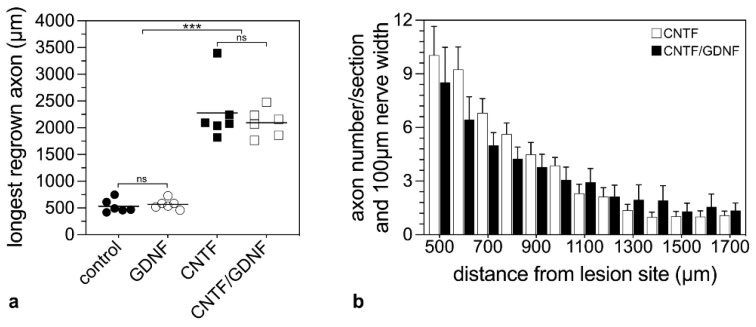
Quantitative analysis of axonal regeneration in animals that had received NSC transplantations 5 days after ONC. The length of the longest regrown axon 28 dpl beyond the lesion site in animals treated with control-NSCs, GDNF-NSCs, CNTF-NSCs or CNTF/GDNF-NSCs (**a**). Each horizontal line represents the mean values of six animals. ns: not significant; ***: *p* < 0.001 according to a one-way ANOVA followed by a Bonferroni post-hoc test. The number of regrown axons in CNTF-treated and CNTF/GDNF-treated mice at different positions distal to the lesion site 28 dpl (**b**). Each bar represents the mean value (±SEM) of six animals. CNTF, ciliary neurotrophic factor; dpl, days post-lesion; GDNF, glial cell line-derived neurotrophic factor; NSC, neural stem cell; ONC, optic nerve crush.

To analyze the effect of the intravitreally administered neuroprotective factors on axonal regeneration in retinas with an advanced RGCs loss, axons were traced anterogradely in animals that had received NSC transplantations 5 dpl (Figure 5). Axonal regeneration was analyzed 28 dpl. In control (Figure 5a) and GDNF-treated animals (Figure 5b), only a few axons were regrown beyond the lesion site. In fact, the longest regrown axons in the control and the GDNF-treated animals extended for only 532.6 ± 50.5 µm (mean ± SEM) and 569.1 ± 37.5 µm, respectively, into the distal nerve stumps (Figure 6a). In comparison, optic nerves of CNTF-treated (Figure 5c) or CNTF/GDNF-treated (Figure 5d) animals contained a markedly greater number of regrown axons that extended for considerable distances beyond the lesion site. The length of the longest regrown axon in CNTF-treated animals was 2278.6 ± 230.0 µm (mean ± SEM), not significantly different from that found in CNTF/GDNF-treated animals (2095.0 ± 105.6 µm; Figure 6a). Importantly, axons followed irregular trajectories and some made U-turns (some marked with arrows in Figure 5c,d) in both experimental groups, indicating that they represent regenerating axons rather than axons that were spared by the lesion.

To compare the number of regenerating axons in CNTF- and CNTF/GDNF-treated animals, we counted the number of labeled axons every 100 µm, starting from 500 µm distal to the lesion site (Figure 6b). Statistical analyses of data revealed similar numbers of regrown axons in both experimental groups at all positions analyzed (Figure 6b). Thus, there was no significant difference in the length of the longest regenerated axons or the number of regenerated axons between CNTF- and CNTF/GDNF-treated animals.

## 4. Discussion

Neuroprotection is being investigated as a potential treatment option for optic neuropathies, including glaucoma. Preclinical studies have identified a number of NTFs that rescue RGCs from cell death in various animal models of optic neuropathies (for reviews, see [17,18,19,60,61]). However, it is often difficult to assess the potential clinical relevance of the results, as many preclinical studies have analyzed RGC survival rates only over a few weeks after treatment initiation and/or have started the neuroprotective treatments at or even before the time RCC degeneration was induced [21,22,31,32,46,52,62,63,64]. However, diseases such as glaucoma typically progress over long periods of time and are usually diagnosed when patients already have significant visual field defects due to the irreversible loss of RGCs and their axons [14,65,66].

We recently found, in a mouse optic nerve crush model, that a co-treatment with CNTF and GDNF synergistically protected intraorbitally lesioned RGCs from cell death, resulting in the survival of approximately 50%, 40% and nearly 40% of the original RGC population 2, 4 and 8 weeks after injury, respectively [45]. Intriguingly, the treatment protected the injured RGCs in the long term. Around 35% of the original RGC population was still viable 8 months after the nerve crush [31]. The observed RGC survival rates are in the range of those reported in studies that have combined a viral vector-mediated delivery of CNTF or GDNF with other pro-survival treatments. For example, in a rat optic nerve transection model, approximately 55% of RGCs were still present 2 weeks after nerve injury and combined the adenoviral delivery of GDNF and the caspase inhibitor X-linked inhibitor of apoptosis (XIAP) [30]. In a mouse optic nerve crush model, AAV2-mediated delivery of CNTF, combined with the conditional deletion of the phosphatase and tensin homolog (PTEN) and suppressor of cytokine signaling 3 (SOCS3) genes, preserved approximately 50% of RGCs over a period of 3 weeks post-lesion [67], and AAV2-mediated delivery of CNTF combined with a peripheral nerve graft sutured to the proximal end of transected optic nerves rescued approximately 25% of RGCs over a period of 7 weeks after injury [21]. In our aforementioned work, we started the co-treatment with CNTF and GDNF shortly after the lesion. Because of the robust and long-lasting RGC rescue observed, in the present study, we investigated the efficacy of the combinatorial neuroprotective treatment in a more clinically relevant situation and started the treatment at a time when a significant proportion of RGCs had already died. We chose 3 and 5 days after ONC as time points to start the treatment, when 25% and 57% of the RGCs were degenerated, respectively. A rapid loss of about 50% of RGCs within 5 days after an intraorbital optic nerve lesion has also been observed by others that have used BRN-3A as a marker to quantify RGCs and has been related to both degeneration of RGCs and downregulation of BRN-3A once RGCs become apoptotic as indicated by the expression of active caspase-3 [68,69].

The half-life of CNTF or GDNF in the vitreous is limited to a few days [70,71]. To provide a continuous supply of NTFs to the retina, we generated CNTF- and GDNF-overexpressing clonal NSC lines and injected the cells into the vitreous cavity. We have shown in previous studies that intravitreally grafted NSCs stop proliferating and rapidly differentiate into neural cell types shortly after transplantation. Furthermore, transplanted cells did not integrate into the retina of recipient animals [50,52]. Consistent with these findings, we did not observe any adverse effects of the transplanted cells on retinal structure in the present study. More importantly, the cells survived in the vitreous cavity and stably expressed the transgenes until the final analysis time point, in agreement with other studies [50,52,59]. While control-NSCs, CNTF-NSCs or GDNF-NSCs differentiated exclusively into astrocytes, we found numerous stem cell-derived neurons in eyes co-treated with CNTF and GDNF. Whether both factors directed the differentiation of the highly neurogenic NSCs [72] into nerve cells, or whether some NSCs spontaneously differentiated into neurons that were then kept alive by the two NTFs, remains to be clarified.

When CNTF and GDNF were co-delivered, the number of surviving RGCs was significantly higher than expected if both NTFs rescued RGCs in an additive manner, regardless of whether the treatment was started 3 or 5 days after ONC. For example, retinas treated with CNTF/GDNF-NSCs 3 dpl and analyzed 56 dpl contained 883 RGCs/mm^2^, 3.3 and 2.7 times more than CNTF- or GDNF-treated retinas, respectively, and approximately 10 times more RGCs than eyes with transplanted control-NSCs. In addition, retinas that were co-treated with both factors 5 dpl and analyzed 56 dpl contained 657 RGCs/mm^2^, representing 38% of the RGC population present at baseline, and 3.5- and 2.7-fold more than in CNTF- and GDNF-treated eyes, respectively. While co-treatment with both NTFs rescued a substantial proportion of the RGC population that had survived to the start of treatment, monotherapy with either CNTF or GDNF had little effect on RGC survival. For example, eyes injected with CNTF-NSCs or GDNF-NSCs 5 dpl and analyzed 56 dpl contained 11% and 14% of the RGC population present at treatment start, respectively. Notably, the number of surviving RGCs in CNTF-treated retinas at this late analysis time point was not significantly different from that in control retinas. An important finding of our previous work was that the combined administration of CNTF and GDNF led to a stabilization of RGC numbers. Remarkably, there was no significant loss of RGCs between consecutive analysis times (i.e., 1, 2, 4, 6 and 8 months after injury) starting from the first month after the lesion, possibly indicating lifelong protection of injured RGCs [31]. In fact, less than 200 RGCs, or about 12% of the RGC population present 1 month after injury died during the 7-month period. In comparison, in the present study, we found a more pronounced loss of about 19% and 14% of RGCs in a much shorter period of time (i.e., between the first and second month after ONC) in animals that received the same treatment, but 3 and 5 days after the lesion, respectively. Results showed that neuroprotective interventions were significantly less effective in slowing RGC degeneration when started at advanced stages of RGC loss.

The synergistic pro-survival effects of the combinatorial neuroprotective treatment are likely mediated by a combination of direct and indirect pathways. RGCs express receptors for CNTF and GDNF [46,73,74,75], and co-administration of both factors may therefore improve RGC survival by activating different pro-survival signaling pathways unique to each factor and by enhancing of pro-survival signaling pathways shared by both factors. In addition, both NTFs may activate indirect neuroprotective pathways more effectively than either factor alone. For example, exogeneous CNTF or GDNF increases endogenous CNTF or GDNF levels and induces retinal glial cells to upregulate and release other NTFs that may further promote RGC survival, such as osteopontin, fibroblast growth factor-2, brain-derived neurotrophic factor or leukemia inhibitory factor [76,77,78,79,80,81]. In addition, both NTFs upregulate the expression of the glutamate/aspartate transporter-1 (GLAST-1) in glial cells, thereby limiting glutamate-related excitotoxicity [20,82].

The extent of axonal regeneration was analyzed 28 dpl by determining the length and number of axons extending beyond the lesion site in animals treated 5 days post-lesion. While CNTF promoted long-distance regeneration with some axons extending more than 2000 µm into the distal nerve stumps along tortuous trajectories, GDNF did not promote axonal regeneration, in line with other reports [83,84,85]. Notably, we found similar numbers of regenerating axons in CNTF- and CNTF/GDNF-treated mice despite the presence of two-fold more surviving RGCs in the latter group at 28 dpl. This finding suggests that CNTF did not stimulate axonal regeneration of RGCs present in the cell population additionally rescued by the co-administration of GDNF. Since the longest axons in control animals extended for more than 700 µm into the distal nerve stump, we consider axons extending more than 800 µm beyond the lesion site as ‘truly regenerated axons’. Interestingly, the number of these axons was essentially the same in animals treated with CNTF either shortly after the lesion [31] or 5 days after the lesion (the present study), even though in the latter group, almost 60% of the normal RGC population had already been lost at the start of treatment, and less than 10% RGCs remained at the time of analysis. This finding suggests that CNTF preferentially stimulates axonal regeneration of injury-resistant RGC subtypes. Indeed, Yungher and colleagues found a few axons extending 1 mm beyond the lesion site in animals that had received intravitreal injections of AAV2-CNTF as late as 56 days after ONC, when only about 4% of RGCs remained [86]. Several studies have shown that intrinsically photosensitive RGCs (ipRGCs) are more resistant to injury than other RGC subtypes [3,7,87,88,89]. A common feature of all ipRGC subtypes (M1-M6) is the expression of the opsin melanopsin (encoded by *Opn4*), which renders these neurons light-sensitive. Bray and colleagues examined ipRGC survival and axonal regeneration following AAV2-mediated CNTF overexpression in a mouse optic nerve crush model by crossing *Opn4*-driven Cre lines with Rosa26-STOP-tdTomato mice [6]. Experiments showed that ipRGCs were more effectively protected from lesion-induced cell death than other RGCs and, more importantly, that a substantial proportion of regenerated axons were tdTomato-positive. Conditional deletion of PTEN also rescued ipRGCs better than other RGCs and promoted regeneration of predominantly tdTomato-positive axons [6]. Taken together, results show that ipRGCs are not only injury-resilient but also, contrary to some previous reports, highly regenerative cells. It is therefore tempting to speculate that ipRGCs contributed substantially to the axonal regeneration observed in the present study, at least partly explaining the similar extent of axonal regeneration after immediate [31] and delayed (the present study) administration of CNTF.

Several studies have demonstrated the feasibility of translating a cell-based intravitreal delivery strategy for NTFs into clinical applications. Renexus^®^ (NT-501; Neurotech Pharmaceuticals, Cumberland, RI, USA) is an intravitreal implant with encapsulated retinal pigment epithelial cells genetically engineered to overexpress CNTF. Clinical trials in patients with geographic atrophy, retinitis pigmentosa or CNGB3-achromatopsia have shown that the NT-501 implants are well tolerated and provide long-term intraocular delivery of the cytokine. However, while the implants preserved retinal structure, they had little or no beneficial effect on retinal function [90,91,92,93,94,95]. The latter observation is probably related, at least in part, to the dysregulation of various genes by the cytokine, including some involved in the phototransduction machinery [96,97,98,99]. However, a randomized, sham-controlled phase 2 trial in patients diagnosed with macular telangiectasia type 2 revealed that, in addition to beneficial effects on retinal structure, loss of retinal sensitivity was attenuated and loss of reading speed was prevented in eyes with NT-501 implants compared to sham-treated eyes [100]. Of interest in the context of the present study, results from an open-label prospective phase I clinical trial in a small group of patients with open-angle glaucoma suggested some structural and functional benefits in eyes treated with the NT-501 device [101].

Important questions to be addressed in future experiments include the effects of the combinatorial treatment on RGC survival in animal models of clinically relevant optic neuropathies, such as glaucoma, and the effects of the treatment on retinal function. Based on the results of the present study, we conclude that combinatorial neuroprotective treatment strategies deserve further research to potentially achieve meaningful therapeutic outcomes. Preclinical studies that initiate neuroprotective interventions after the onset of neurodegenerative retinal diseases and monitor treatment outcomes over as long a period as possible will help to better assess the relevance of neuroprotective treatment strategies for potential clinical applications.

## Data Availability

The data presented in this study are available on request from the corresponding author.

## Supplementary Materials

The following supporting information can be downloaded at: https://www.mdpi.com/article/10.3390/cells14090643/s1, Figure S1: The impact of the delay between ONC and NSC transplantation at different post-lesion time points; Figure S2: Comparison of the efficacy of neuroprotective treatments initiated 3 dpl or 5 dpl; Figure S3: Retinal morphology of animals treated with CNTF/GDNF-NSCs or control-NSCs; Table S1. Densities of retinal ganglion cells in experimentally treated animals.
