# Supplementary material for: Survival and Axonal Regeneration of Retinal Ganglion Cells in a Mouse Optic Nerve Crush Model After a Cell-Based Intravitreal Co-Administration of Ciliary Neurotrophic Factor and Glial Cell Line-Derived Neurotrophic Factor at Different Post-Lesion Time Points
Source: Cells. 2025 Apr 28;14(9):643. doi: 10.3390/cells14090643 (PMC12071274; doi:10.3390/cells14090643)
Supplement: Supplementary file 1 [file cells-14-00643-s001.zip › cells-3540712-supplementary.pdf]

Supplementary Materials

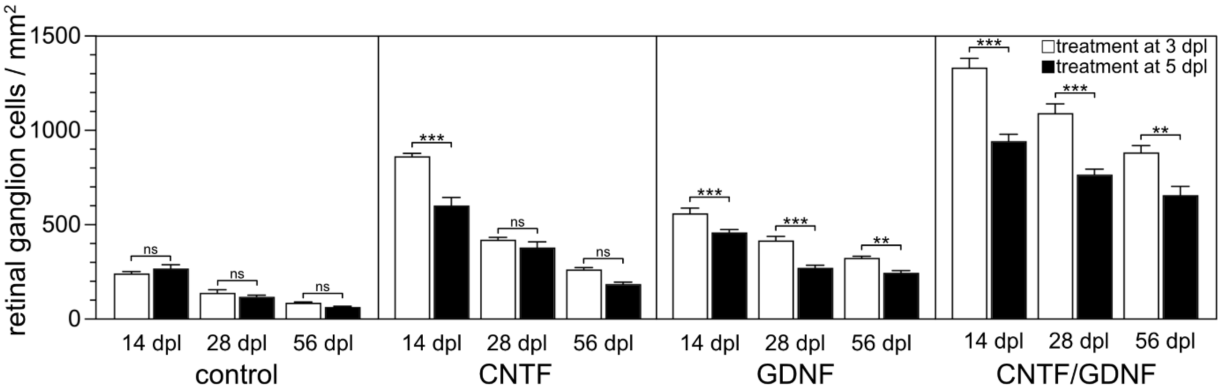

Figure S1. The impact of the delay between ONC and NSC transplantation on the progression of RGC loss at different post-lesion time points. RGC densities in animals that received transplantations of control-NSCs either 3 or 5 days after ONC were similar at each time point analyzed. RGC densities in retinas treated with the different neurotrophic factors, in comparison, were significantly lower at each analysis time point when the cell transplantation was performed 5 dpl as opposed to 3 dpl, with the sole exception of CNTF-treated retinas 28 dpl and 56 dpl. Each bar represents the mean value (±SEM) of six animals. ns: not significant; **: $p < 0.01$; ***: $p < 0.001$ according to a two-way ANOVA followed by a Bonferroni post-hoc test. CNTF, ciliary neurotrophic factor; dpl, days post-lesion; GDNF, glial cell line-derived neurotrophic factor; NSC, neural stem cell; ONC, optic nerve crush; RGC, retinal ganglion cell.

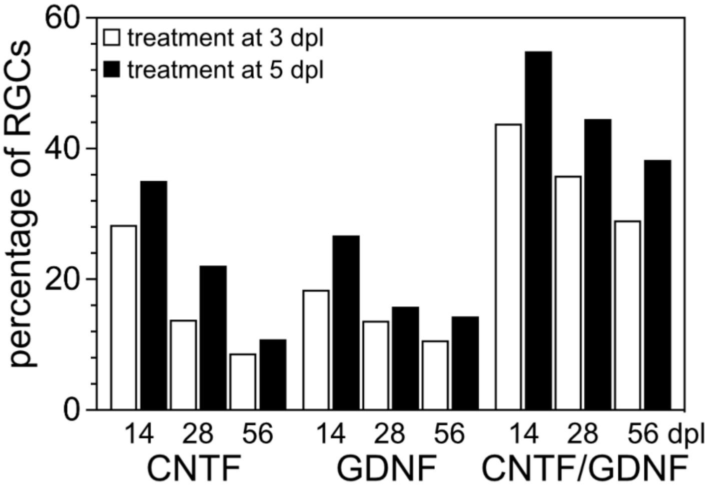

Figure S2. Comparison of the efficacy of neuroprotective treatments initiated 3 dpl or 5 dpl.
Treatments initiated 5 dpl rescued a higher percentage of the RGCs population present at the time of NTF administration than treatments initiated 3 dpl. CNTF, ciliary neurotrophic factor; dpl, days post-lesion; GDNF, glial cell line-derived neurotrophic factor; RGCs, retinal ganglion cells.

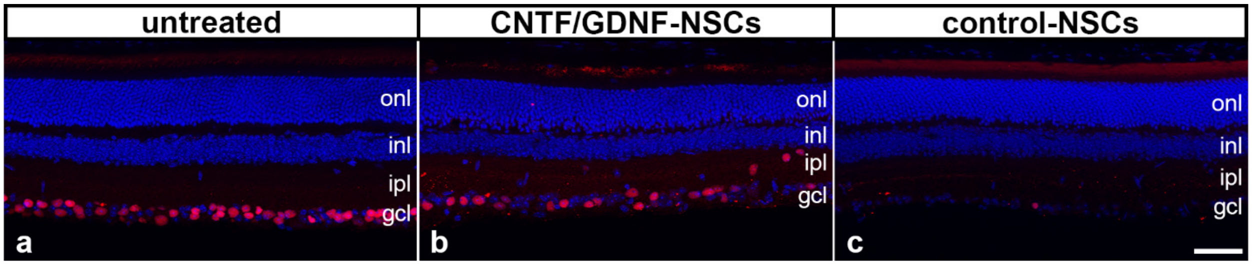

Figure S3. Retinal morphology of animals treated with CNTF/GDNF-NSCs or control-NSCs.
Retinal sections from animals that received neither an optic nerve crush nor cell transplantation (**a**) or from animals treated with CNTF/GDNF-NSCs (**b**) or control-NSCs (**c**) 5 days post-lesion and sacrificed 56 days after the lesion were stained with antibodies against BRN-3A. Cell nuclei were stained with DAPI to visualize retinal layers. Comparison of untreated (**a**) and treated (**b**,**c**) retinas shows that the treatment had no adverse effect on retinal morphology. BRN-3A-positive ganglion cells were numerous in retinas treated with CNTF/GDNF-NSCs (**b**), but almost absent in retinas treated with control-NSCs (**c**). BRN-3A, brain-specific homeobox/POU domain protein 3A; gcl, ganglion cell layer; inl, inner nuclear layer; ipl, inner plexiform layer; onl, outer nuclear layer; NSC, neural stem cell. Scale bar: 50 µm.

**cell transplantation 3 days after ONC**					
**analysis**	**control**	**CNTF**	**GDNF**	**CNTF + GDNF - control**	**CNTF/GDNF**
**14 dpl**	242.0	862.7	561.0	1,181.7	1,333.7
**28 dpl**	139.3	420.3	416.3	697.3	1,092.0
**56 dpl**	86.3	263.7	324.8	502.2	883.0
**cell transplantation 5 days after ONC**					
**analysis**	**control**	**CNTF**	**GDNF**	**CNTF + GDNF - control**	**CNTF/GDNF**
**14 dpl**	267.8	602.2	458.8	793.2	942.5
**28 dpl**	117.5	379.3	272.2	534.0	765.0
**56 dpl**	64.3	186.0	245.7	367.4	657.2

Table S1. Densities of retinal ganglion cells in experimentally treated animals.
The values indicate the density of retinal ganglion cells per mm^2 of retinal area at different time points after an optic nerve lesion (i.e. 14, 28 or 56 dpl) in animals that received intravitreal transplantations of control-NSCs, CNTF-NSCs, GDNF-NSCs or a 1:1 mixture of CNTF- and GDNF-NSCs either three or five days after ONC. To determine whether co-adminstration of CNTF and GDNF promoted RGC survival in a more than additive manner, values of animals that were treated with either CNTF or GDNF were added, and the values of the corresponding controls were subtracted to not consider those RGCs twice that would have survived without treatment. A comparison of the calculated RGC densities (CNTF + GDNF – control) with those found in retinas co-treated with CNTF and GDNF (CNTF/GDNF) shows that the co-administration of both neurotrophic factors promoted RGC survival in a synergistic manner in all experimental groups, regardless of whether the cell transplantations were performed three or five days after the nerve lesion. Each value represents the mean of six animals. dpl, days post-lesion; ONC, optic nerve crush.
